# Transcriptomics-guided optimization of vitamins to enhance erythromycin yield in *saccharopolyspora erythraea*

**DOI:** 10.1186/s40643-024-00817-w

**Published:** 2024-11-01

**Authors:** Xiang Ke, Xing Jiang, Shuohan Wang, Xiwei Tian, Ju Chu

**Affiliations:** 1 State Key Laboratory of Bioreactor Engineering, East China University of Science and Technology, 130 Meilong Road, Shanghai, 200237 China; 2 Qingdao Innovation Institute of East China University of Science and Technology, 596-1 East Jiushui Road, Qingdao, 266102 China

**Keywords:** Erythromycin, Vitamin, *Saccharopolyspora erythraea*, Transcriptomics, Metabolomics, Metabolic flux analysis

## Abstract

Comparative transcriptomics uncovered distinct expression patterns of genes associated with cofactor and vitamin metabolism in the high-yielding mutant strain *Saccharopolyspora erythraea* HL3168 E3, as compared to the wild-type NRRL 2338. An in-depth analysis was conducted on the effects of nine vitamins, and it was determined that thiamine pyrophosphate (TPP), vitamin B2, vitamin B6, vitamin B9, vitamin B12, and hemin are key enhancers in erythromycin production in E3, increasing the erythromycin titer by 7.96–12.66%. Then, the Plackett-Burman design and the path of steepest ascent were applied to further optimize the vitamin combination for maximum production efficiency, enhancing the erythromycin titer in shake flasks by 39.2%. Otherwise, targeted metabolomics and metabolic flux analysis illuminated how vitamin supplementation modulates the central carbon metabolism with notable effects on the TCA cycle and methionine synthesis to augment the provision of energy and precursors essential for erythromycin synthesis. This work highlights the capacity for precise vitamin supplementation to refine metabolic pathways, thereby boosting erythromycin production, and provides valuable directions for application on an industrial scale.

**Graphical Abstract:**

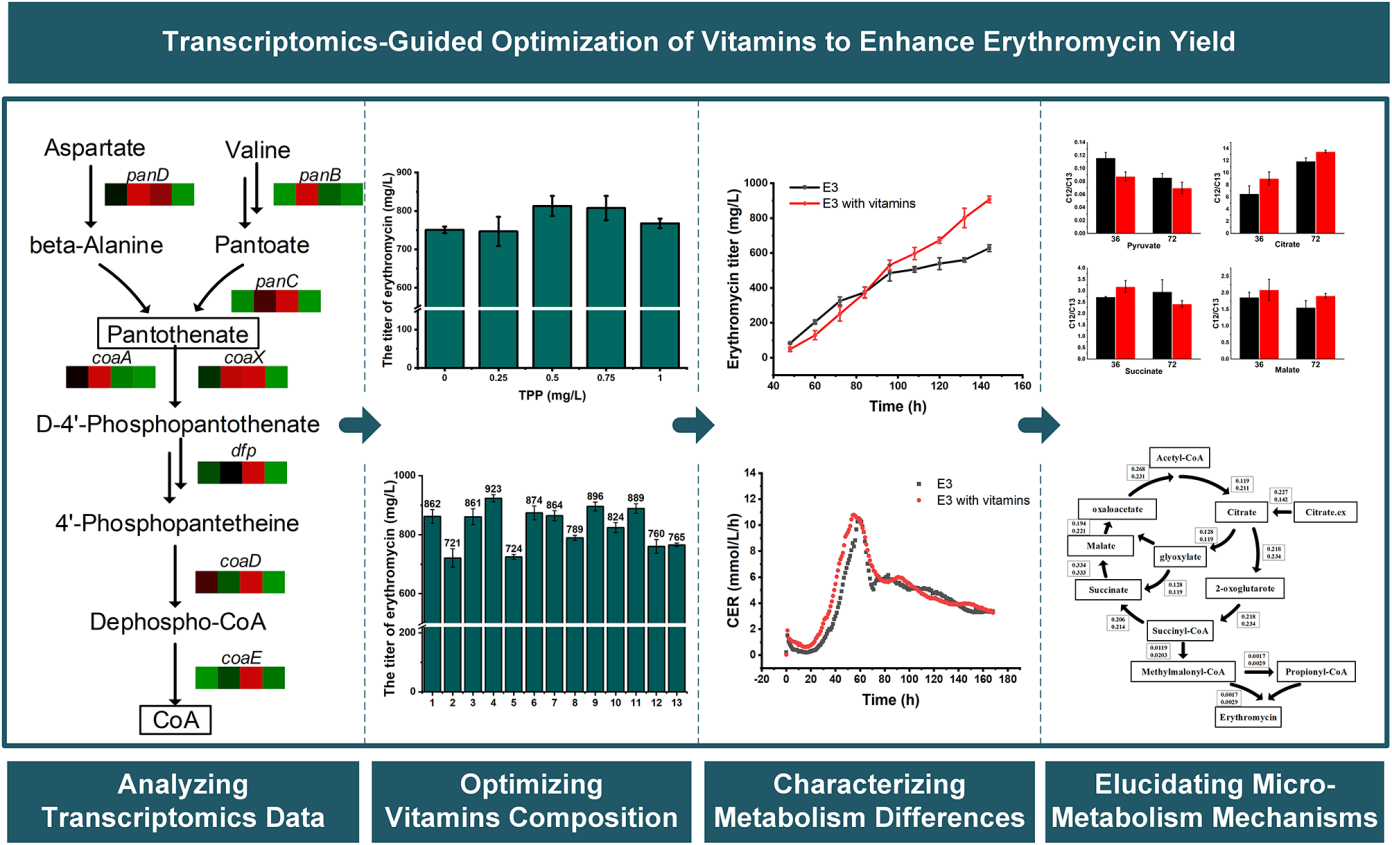

**Supplementary Information:**

The online version contains supplementary material available at 10.1186/s40643-024-00817-w.

## Introduction

Actinobacteria, a significant bacterial order, are prolific producers of antibiotics and other valuable compounds (Palazzotto et al. 2019). Despite their potential, wild-type strains often produce insufficient quantities of desired products, prompting the need for extensive mutagenesis and cultivation optimization to increase titers. The complex regulatory networks in actinomycetes, due to their larger genomes, make it challenging to enhance the production of secondary metabolites (Redenbach et al. 2000). Recent advances in DNA and RNA sequencing, along with the development of genetic tools, have facilitated more effective metabolic engineering approaches. These methods, such as multi-omics analysis and reverse metabolic engineering, have been key in identifying factors that can boost the production of secondary metabolites (Weber et al. 2015; Bu et al. 2021).

*Saccharopolyspora erythraea*, a member of the actinobacteria group, holds a pivotal role in the pharmaceutical industry as a key producer of erythromycin. Erythromycin features a distinctive macrocyclic lactone ring adorned with desosamine sugars, exerts its bactericidal effect by specifically targeting the 50 S subunit of bacterial ribosomes. This antibiotic is valued for its extensive antimicrobial spectrum and widespread application in various treatments (Park and Yoon 2019). Previously, the majority of efforts to increase erythromycin production have centered on genetic manipulations designed to enhance the expression of biosynthesis gene cluster or the synthesis of erythromycin precursors (Guo et al. 2016; You et al. 2019; Jiang and Pfeifer 2013). The complete genome sequencing of *S. erythraea* NRRL2338 in 2007 initiated a new chapter in comparative omics for the species(Oliynyk et al. 2007). These omics-based comparisons have shed light on the profound features, such as transcription regulators and cofactors, that may be linked to the high-producing phenotype of erythromycin (Peano et al. 2014; Li et al. 2013). However, despite these insights, the strategies proposed and confirmed to enhance erythromycin production is still quite limited. The challenge lies in translating the wealth of omics data into feasible engineering strategies that can effectively enhance erythromycin production.

Cofactors are indispensable to the function of enzyme proteins, playing a critical role in the transfer of electrons, atoms, and chemical groups (Wang et al. 2017). Water-soluble B-complex vitamins and their active forms are particularly crucial as cofactors in intracellular metabolic pathways (Depeint et al. 2006). They serve to catalyze the precise transfer of specific molecular components between metabolites, thereby facilitating the metabolism of proteins, lipids, and carbohydrates. Adjusting intracellular vitamin levels has been proven to exert a significant influence on enzyme activities, bacterial growth, and the overall metabolic processes. Strategies that aim to modify the biosynthetic capacity of intracellular vitamins or to manipulate their exogenous supply are essential for regulating these levels (Huang et al. 2006; Liao et al. 2018). However, there is a notable absence of systematic research on the impact of B vitamins on erythromycin production to date.

In this study, the comparative transcriptomic analysis had uncovered distinctive expression disparities in genes related to vitamin and cofactor metabolic pathways between the *S. erythraea* E3 and NRRL2338 strains. These differences suggested that regulating the levels of intercellular vitamins or cofactors might potentially facilitate the production of erythromycin in E3. To further investigate the precise influence of vitamins on erythromycin biosynthesis, nine vitamins were added exogenously individually or in combination into a chemically defined medium. And then the 5 L fermentation and intracellular metabolite profiling were carried out. Finally, metabolic flux analysis was utilized to dissect the potential effects of these vitamins on the central carbon metabolic pathways in *S.erythraea* E3.

## Materials and methods

### Strains

Two *S. erythraea* strains were employed in this study. The *S. erythraea* HL3168 E3 strain was developed from the wild-type NRRL2338 (ATCC 11,635) using traditional mutation and screening methods.

### Media and culture conditions

XM solid medium (per liter): starch 10.0 g, corn steep liquor 10.0 g, NaCl 3.0 g, (NH_4_)_2_SO_4_ 3.0 g, CaCO_3_ 5.0 g, agar 20.0 g, pH 7.0.

Seed medium (per liter): starch 40 g, peptone 20 g, NaCl 4 g, glucose 10 g, KH_2_PO_4_ 0.2 g, CaCO_3_ 6 g, MgSO_4_ 0.25 g, pH 7.0.

Chemically defined fermentation medium (per liter): glucose 22 g, sodium citrate 2.28 g, K_2_HPO_4_ 1.28 g, KH_2_PO_4_ 0.64 g, MgSO_4_·7H_2_O 1 g, alanine 0.86 g, arginine 0.68 g, cysteine 0.78 g, serine 0.73 g, and trace element stock solution 10 mL, pH 7.0.

100×Trace element stock solution (per liter): CoCl_2_ 0.9 g, Na_2_B_4_O_7_ 0.6 g, FeCl_3_ 0.68 g, CuCl_2_ 0.027 g, (NH_4_)_2_MoO_4_ 0.027 g.

For the induction of sporulation in *S. erythraea* strains, cultures were grown on XM solid medium at a constant temperature of 34 °C for a duration ranging from 5 to 7 days. The harvested spores were then inoculated into a seed medium within a 500-mL shake flask, initiating with a volume of 50 mL and subjected to incubation conditions of 34 °C and 220 rpm. Following a 48-hour period of pre-cultivation, an aliquot of 5 mL from the seed culture was introduced into 500-mL shake flasks containing 45 mL of a chemically defined fermentation medium. The inoculation method for 5 L bioreactor culture adhered to a standardized protocol, with an inoculum volume constituting 10% of the total culture volume. The DO was controlled higher than 30% by adjusting the aeration rate and the stirring speed. The pH was maintained at 7.0 with the addition of NaOH and the temperature was held at 34 °C.

### Design of single factor experiments

Single-factor experiments were conducted to ascertain the impact of various cofactors on erythromycin yield and cell growth. The concentration ranges for the cofactors were selected based on findings from prior studies (Yu et al. 2011). The experimental levels for each cofactor were as follows: TPP at 0–1 mg/L, VB_2_ at 0–1.125 mg/L, VB_3_ at 0–6 mg/L, calcium VB_5_ at 0–6 mg/L, VB_6_ at 0–0.6 mg/L, VB_7_ at 0–0.15 mg/L, VB_9_ at 0–10 mg/L, VB_12_ at 0–0.6 mg/L, and hemin at 0–16 mg/L. Each vitamin was subjected to micro-filtration for sterilization and then incorporated into the fermentation medium as per the experimental design. All the vitamins were purchased from Aladdin.

### Plackett–Burman design

The Plackett-Burman design was used to determine the most effective vitamins composition, employing the Design-Expert software (Version 12.0, USA). Variables were examined over two levels, designated as high (+ 1) and low (-1). The specific levels for each factor were detailed in Table S1. A total of 13 experimental runs were devised to assess the impact of six variables, as presented in Table S2. Triplicate measurements were taken for all trials to ensure accuracy. Subsequent to the experiments, Plackett–Burman data were subject to the F-test, where the estimated effect, sum of squares, and F-value were calculated according to the methods described by Analytical Methods Committee Technical Briefs (AMCTB).

### Determination of the path of steepest ascent

According to the initial regression equation derived from the outcomes of the Plackett-Burman test, the trajectory for the path of steepest ascent was delineated Table 1. The direction of adjustment for each factor is indicated by the sign of its coefficient: a negative coefficient suggests a reduction in the factor level, and a positive coefficient implies an increase. Adhering to the path of steepest ascent experimental design, the optimal fermentation parameters were established to maximize erythromycin yield.

**Table 1:** Plackett-Burman Design Statistical Analysis

	Sum of		Standardized	F	*p*-value	
Source	Squares	df	Effects	Value	Prob > F	
Model	46627.23	6		10.53	0.0103	significant
A-TPP	2460.37	1	31.50	3.33	0.1274	
B-B2	25453.97	1	-101.32	34.5	0.002	**
C-B6	6302.69	1	-50.42	8.54	0.0329	*
D-folate	3148.47	1	35.64	4.27	0.0937	
E-B12	8703.95	1	59.25	11.8	0.0185	*
F-hemin	557.78	1	15.00	0.756	0.4243	
Curvature	4835.31	1		6.55	0.0506	
Residual	3688.94	5				
Cor Total	55151.48	12				

The model F-value = 10.53 indicates that the model is significant. P-value < 0.0500 suggests that the model terms are significant. * *p* < 0.05; ** *p* < 0.01; *** *p* < 0.001

### Determination of intracellular metabolite pools

The measurements of intracellular metabolite pools were conducted following the protocol outlined in our preceding research (Hong et al. 2017).

### Metabolic flux analysis

We employed the metabolic flux analysis model developed by Xu et al. (Xu et al. 2021), incorporating the rates of glucose and oxygen consumption alongside erythromycin and carbon dioxide production into our analysis, as presented in Table S3.

## Results and discussion

### Comparative transcriptomics revealed distinct expression patterns of genes involved in vitamin and cofactor metabolism

**Fig. 1:**
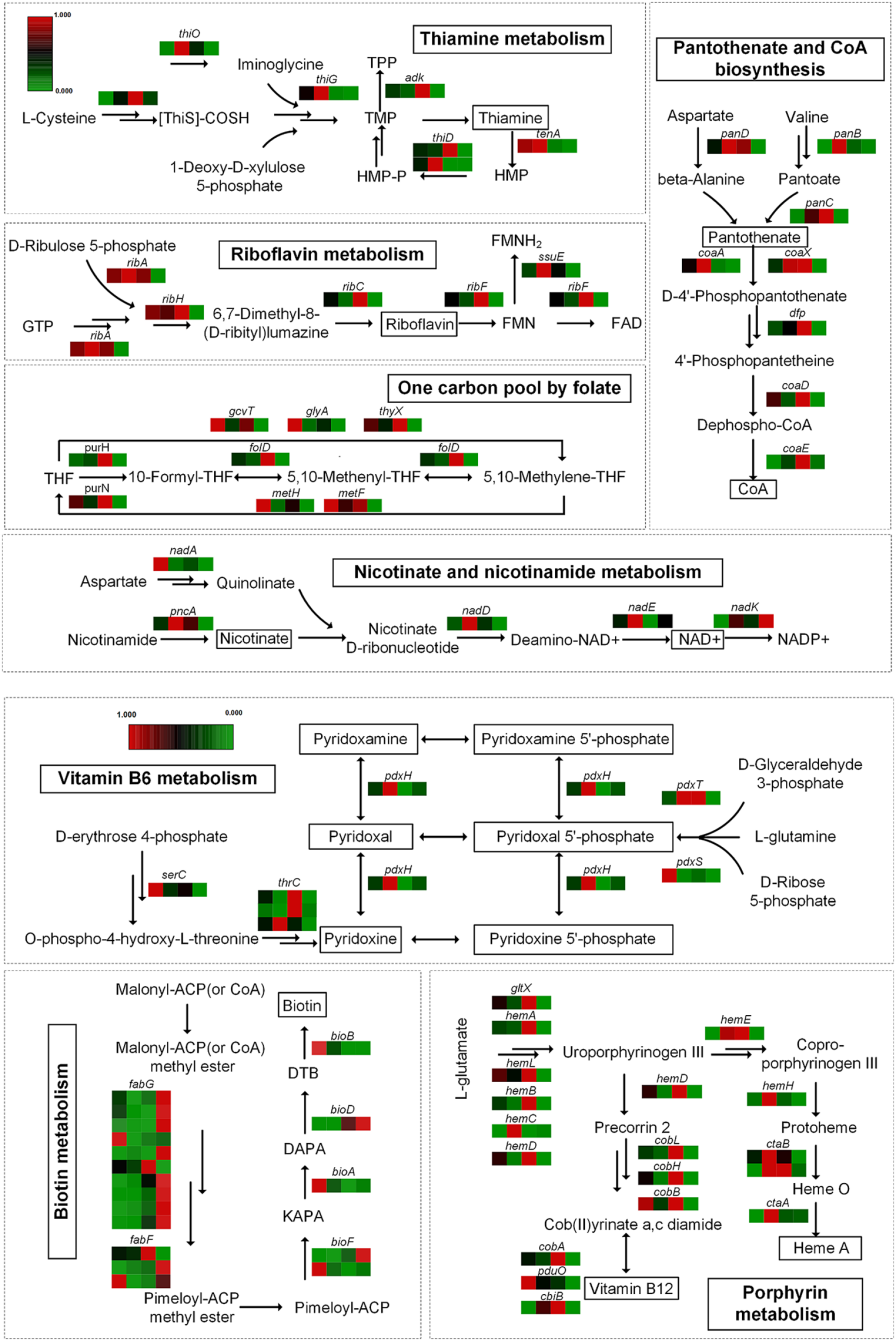
Comparative transcriptional analysis of vitamin metabolism between *S. erythraea* E3 and WT. The colored rectangular bars adjacent to the black arrows illustrate the normalized gene expression levels for the genes involved in the metabolic reactions. The order of the bars from left to right represents the WT-10 h, WT-50 h, mutant E3-10 h, and E3-50 h, respectively

To reveal the genetic basis of the high erythromycin producing mutant *S. erythraea* HL3168 E3, we compared its gene expression patterns over time with those of wild type strain NRRL 2338 in our previous study (Li et al. 2022). Differential gene expression analysis and KEGG enrichment analysis revealed specifically upregulation of genes expression related to cofactor and vitamin metabolism during the erythromycin high-yield phase of E3 (Fig. S1 and S2). Based on that, we normalized and systematically classified the transcriptional profiles of the implicated genes. KEGG pathway analysis uncovered that these genes were primarily involved in the synthesis pathways of B vitamins, including thiamine metabolism, riboflavin metabolism, pantothenic acid and CoA biosynthesis, one carbon pool by folate, nicotinate and nicotinamide metabolism, vitamin B_6_ metabolism, biotin metabolism, and porphyrin metabolism (Fig. 1). A considerable subset of these genes, pivotal to the synthesis and metabolism of B vitamins, displayed heightened expression during the stationary phase of E3. In the context of porphyrin metabolism, the genes responsible for synthesizing the heme vitamin, in addition to cobalamin, were also found to be upregulated during this phase. In contrast, a subset of genes within the biotin synthesis pathway was found to be significantly downregulated at the transcriptional level (Fig. 1), especially the expression levels of multiple isoenzyme genes of fabG and fabF during the E3 production phase (Shanbhag 2019). Since vitamins were important sources of various cofactors within the cell, changes in the expression of these genes might affect the concentration of intracellular vitamins and cofactors, thereby altering the growth and secondary metabolic characteristics of *S. erythraea*. Given the complexity of vitamin synthesis, adding exogenous vitamins might be the most direct and effective strategy to explore their impact on erythromycin synthesis.

### Individual addition of vitamins affected the erythromycin yield of E3 in chemically defined medium

**Fig. 2:**
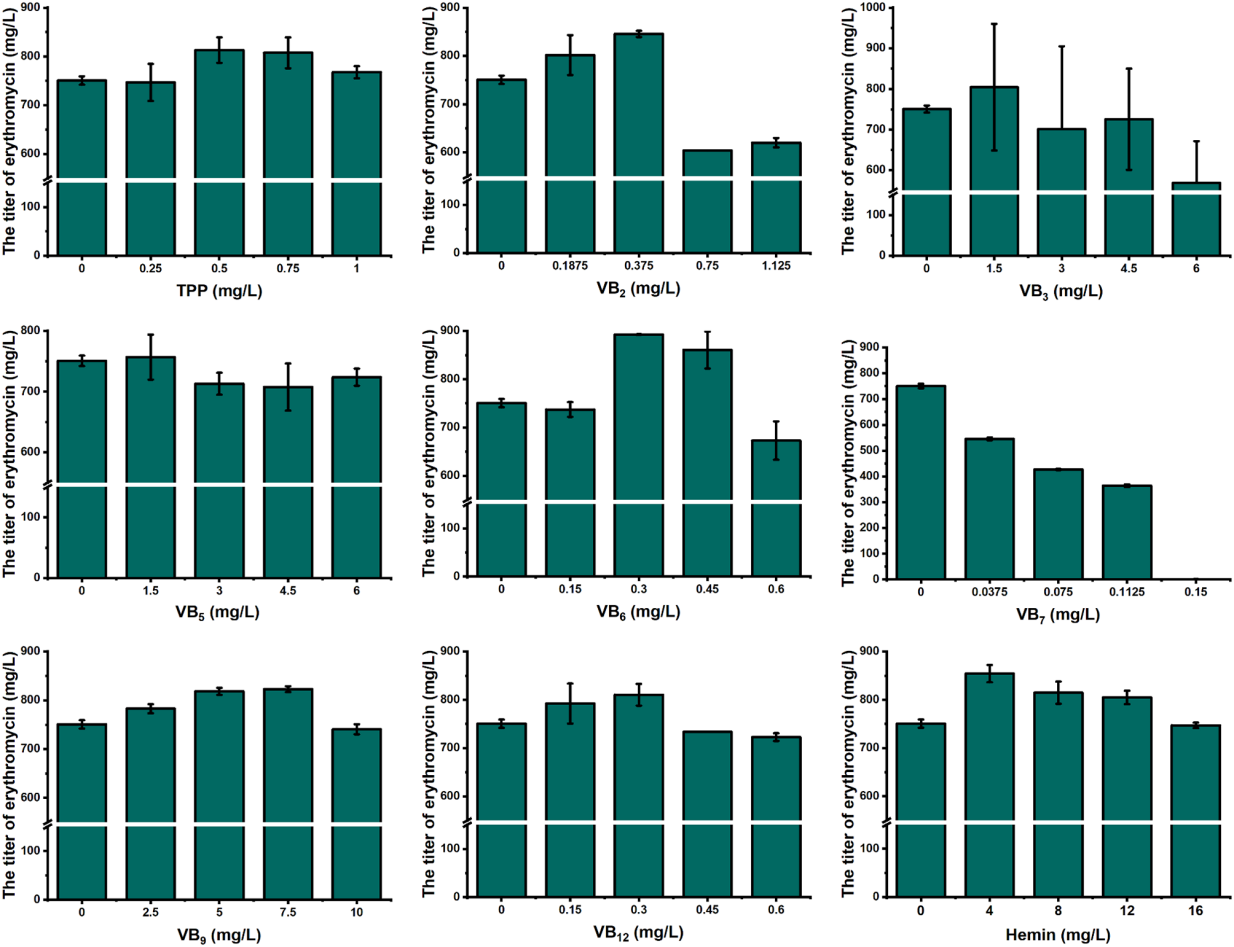
The impact of vitamin gradient supplementation on erythromycin biosynthesis

Drawing on the findings from the transcriptome analysis, we undertook an investigation to assess the influence of vitamin supplementation on erythromycin yield. A chemically defined medium was utilized, ensuring a transparent composition of its components and minimizing interference, thereby facilitating a more accurate analysis. Following a similar approach to a previous study with *Streptomyces roseosporus*,we initiated fermentation with the addition of nine different vitamins or their derivatives, including TPP, VB_2_, VB_3_, VB_5_, VB_6_, VB_7_, VB_9_, VB_12_, and hemin, at varying concentrations to the medium(Yu et al. 2011). The results indicated that six of them, TPP, VB_2_, VB_6_, VB_9_, VB_12_, and hemin, positively influenced erythromycin synthesis, culminating in an increase in erythromycin yield by 7.96–12.66% (Fig. 2). Conversely, the addition of VB_3_ induced instability in erythromycin synthesis, possibly leading to the accumulation of by-products and a consequent decline in erythromycin production. In the case of VB_7_, the addition led to a pronounced inhibition of erythromycin production. An increase in the concentration of biotin within the medium was correlated with a decrease in erythromycin yield, with a concentration of 0.15 mg/L of biotin causing significant inhibition of cell growth and a blockade in erythromycin synthesis. It was worth emphasizing that the outcomes from the vitamin supplementation experiment correlated well with the transcription analysis. Specifically, six of the eight vitamins, whose synthesis-related genes exhibited upregulation, were verified to boost erythromycin synthesis. In contrast, biotin, with its related genes showing downregulation, was found to hinder erythromycin synthesis. This comparison emphasized the consistency between gene expression patterns and the functional effects of vitamins on erythromycin production.

Given the known physiological roles of vitamins, it was possible to hypothesize the mechanisms by which vitamin supplementation might influence erythromycin production. TPP, a coenzyme for pyruvate dehydrogenase and alpha-ketoglutarate dehydrogenase, might positively affect central carbon metabolism, potentially promoting the utilization of carbon sources and the supply of erythromycin precursors (Bettendorff and Wins 2009). Flavin mononucleotide (FMN) and flavin adenine dinucleotide (FAD), the active forms of VB_2_, served as cofactors for some oxidoreductases, such as succinate dehydrogenase and NADH dehydrogenase (Averianova et al. 2020). VB_6_ might facilitate the assimilation of amino acids in the synthetic medium, while VB_9_ aided in the transfer of one-carbon units and the de novo synthesis of pyrimidines and purines, potentially enhancing erythromycin yield by promoting cell growth and optimizing methyl group supply (Rosenberg et al. 2017; Phillips 2015; Selhub 2002). The enhancement of energy metabolism could be the possible reason why VB_2_ was beneficial for erythromycin production. VB_12_ was involved in methyl transfer reactions and acts as a coenzyme for several mutases, including methylaspartate mutase and methylmalonyl-CoA mutase, which could disrupt the supply of precursors and methyl groups, leading to an increase in erythromycin production (Froese et al. 2019). Hemin, a complex of iron porphyrin, was a prosthetic group for proteins like myoglobin, catalase, and cytochromes (Layer 2021). The potential mechanism by which hemin affected erythromycin synthesis might involve boosting cellular oxygen absorption and utilization, enhancing intracellular redox reactions, and impacting intracellular energy levels. On the other hand, biotin, a coenzyme for intracellular carboxylases, including acetyl-CoA carboxylase, linked glycolysis with fatty acid synthesis pathways (León-Del-Río 2019). The addition of biotin might enhance the activity of acetyl-CoA carboxylase, directing more acetyl-CoA towards fatty acid synthesis and away from the TCA cycle. This shift might decrease the availability of erythromycin precursors, hinder cellular energy metabolism, inhibit cell growth, and ultimately, reduce erythromycin yield.

### Optimization of combined vitamin supplementation enhanced erythromycin production

The supplementation of specific vitamin had been shown to enhance the erythromycin biosynthetic capacity of E3. However, previous studies had also pointed out that vitamins often present synergistic effects, which could interact to regulate biosynthetic pathways and collaboratively influence biological functions (Lyon et al. 2020). For instance, VB_2_ was involved in the metabolic pathways of VB_3_ and VB_6_, with its active derivatives, FAD and FMN. VB_12_ enhanced the efficacy of VB_9_, participating in the synthesis of methionine from homocysteine and the production of choline. Without VB_12_, the transfer of methyl groups was hindered, making VB_9_ unusable for metabolism, which might cause a VB_9_ deficiency (Selhub 2002; Froese et al. 2019). Given the complex interactions between vitamins, combined supplementation might affect the optimal concentration to achieve maximum production capacity. Then, Plackett-Burman design (PBD) was continually conducted.

Based on the results of the initial experiment, a two-level PBD of 13 runs was implemented for 6 vitamins known to enhance erythromycin yield. Table S1 and S2 outlined the input variable and their levels in PBD. Each estimated variables were examined in two levels, low (−) and high (+) level. The corresponding erythromycin production of each run was measured and the ANOVA was applied to analysis the result of PB model (Fig. S3). The F-value of the model was 10.53, indicating the model was significant overall. The p-value of VB_2_, VB_6_ and VB_12_ were under 0.05, suggesting these vitamins were major factors to influence erythromycin production (Table 1). Otherwise, the model showed that the erythromycin yield was correlated with various components, represented by the equation: C_Ery_(µg/mL) = 832.65 + 14.32*TPP-46.06*VB_2_ − 22.92*VB_6_ + 16.20*VB_9_ + 26.93*VB_12_ + 6.82*Hemin. The coefficients in this equation indicated the impact of each component on erythromycin production, with the degree of impact correlating to the coefficients’ absolute values. The findings suggested that vitamins B_2_, B_6_, and B_12_ were likely the principal vitamins in erythromycin synthesis.

To further optimize vitamin supplementation composition, an experimental design following the path of steepest ascent was conducted, focusing on VB_2_, VB_6_, and VB_12_. The adjustment of the concentration of the vitamins was based on the positive or negative effects of component estimation coefficients. Among them, VB_2_ and VB_6_ had negative coefficient so that their addition concentration should be reduced while VB_12_ showed positive coefficient, indicating a need to increase its concentration. The vitamin concentration of each combination and corresponding erythromycin production were shown in Table 2. In this study, the peak erythromycin production was achieved in group 5, with a combination of 0.15 mg/L VB_2_, 0.21 mg/L VB_6_ and 0.39 mg/L VB_12_. This vitamin combination notably enhanced the erythromycin yield in shake flasks by 39.2% over the baseline production of 724.8 mg/L without vitamin addition.

**Table 2:** The construction and results of the steepest ascent experiment

*N*	VB_2_ (mg/L)	VB_6_ (mg/L)	VB_12_ (mg/L)	Erythromycin titer (mg/L)
1	0.4125	0.3300	0.2700	972.49
2	0.3750	0.3000	0.3000	902.38
3	0.3000	0.2700	0.3300	948.24
4	0.2250	0.2400	0.3600	944.19
5	0.1500	0.2100	0.3900	1008.61
6	0.0750	0.1800	0.4200	1005.71

### Combined supplementation of vitamins influenced the fermentation properties of E3 in 5 L bioreactor

**Fig. 3:**
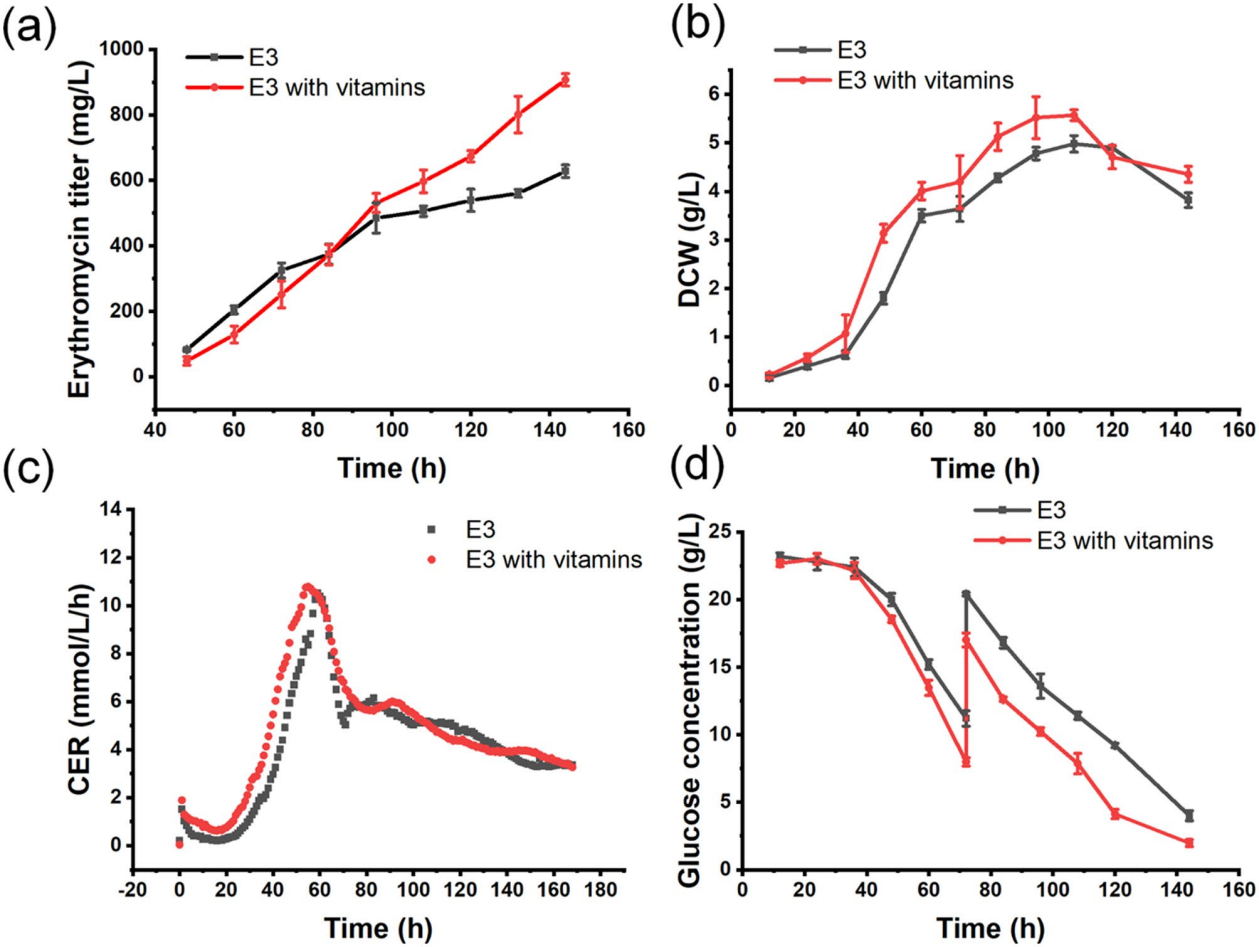
Time course of process parameters for *S. erythraea* E3 and vitamin-supplemented E3 in a 5 L bioreactor. (**a**) Erythromycin titer (**b**) Dry cell weight (DCW) (**c**) Carbon dioxide emission rates (CER) (**d**) Residual glucose concentration. Data ± SE; *n* = 3

After that, we delved into the physiological effects of vitamin supplementation on erythromycin production by quantifying the metabolic characteristics of E3 in a 5 L bioreactor. The results demonstrated that the erythromycin concentration in the experimental group reached to 907.1 mg/L at 144 h, marking a significant 44.4% increase over the control group (Fig. 3a). DCW curve revealed that the initial fermentation phase was enhanced by vitamin supplementation (Fig. 3b). The experimental group exhibited a pronounced early surge in the CER, which began and peaked 6 h ahead of the control group. In the stationary phase of fermentation process, the CER levels between the experimental and control groups were notably similar, indicating a stabilization of metabolic activity (Fig. 3c). Glucose consumption patterns were parallel for both groups, however, the experimental group showed an accelerated rate of glucose consumption during the early stage of fermentation (Fig. 3d). Thus, it was deduced that vitamin supplementation expedited glucose utilization, potentially improving the central carbon metabolic pathways. This enhancement could enhance the erythromycin synthesis capability by increasing the availability of energy and precursor molecules. Moreover, the experimental group sustained a consistent erythromycin accumulation rate throughout the fermentation process, while the control group experienced a decline in erythromycin production after 96 h. This finding suggested that vitamins play a crucial role in maintaining erythromycin synthetic metabolism in the stationary phase, and enhanced the conversion rate of glucose to erythromycin.

### Targeted metabolomics revealed the impact of vitamins on intracellular metabolites concentration

**Fig. 4:**
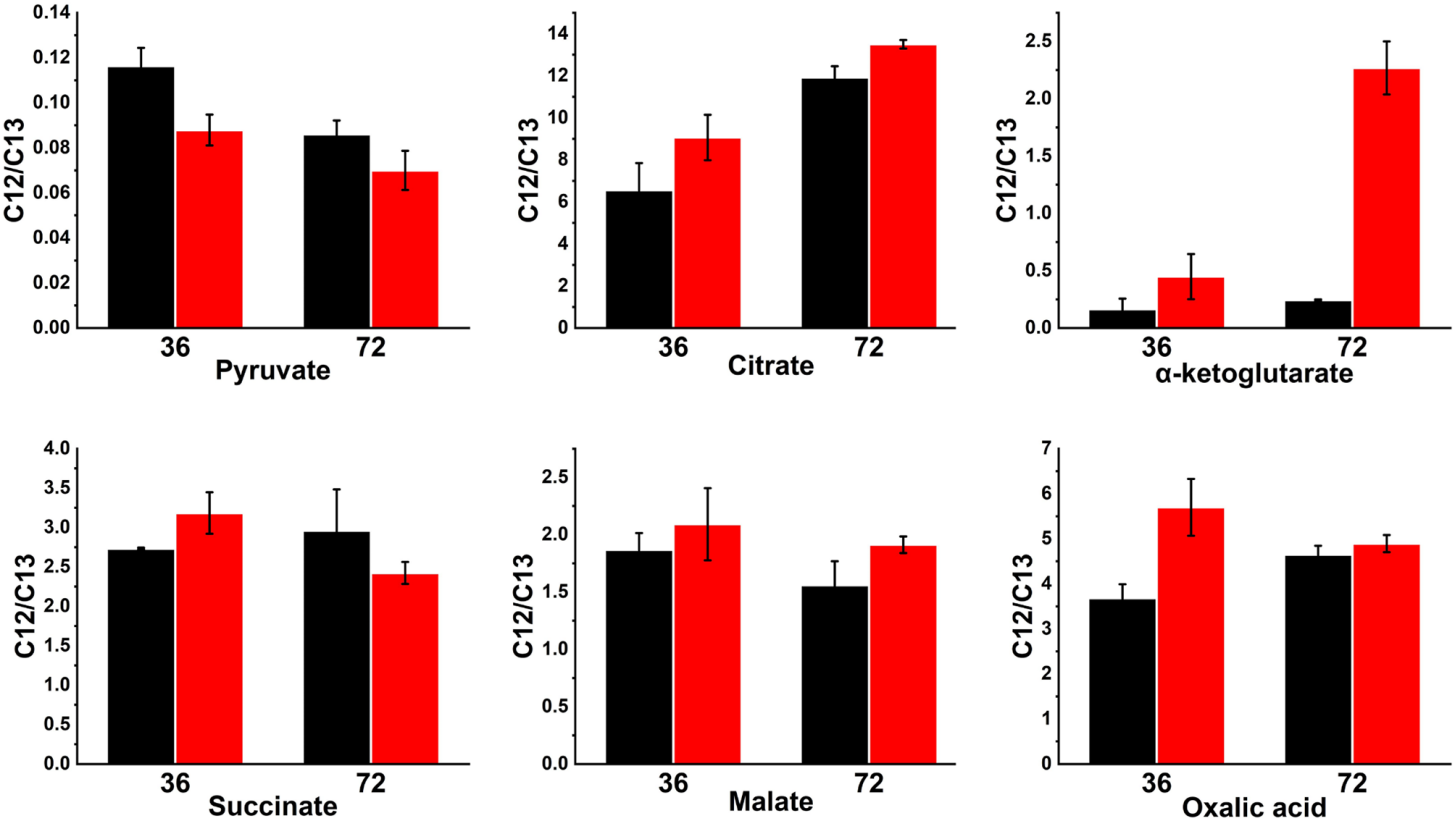
The effects of vitamins supplementation on the relative intracellular concentrations of central carbon metabolites in E3 at 36 h and 72 h. The relative concentration of intracellular organic acids is represented by the ratio of ^12^C intracellular metabolites to ^13^C internal standards

We further attempted to explore the effects of vitamin supplementation from a microscopic metabolic perspective. Isotope dilution mass spectrometry (IDMS)-based targeted metabolomics was introduced to identify the relative levels of intracellular organic acids and amino acids in conditions with and without adding vitamins. The relative concentrations of metabolites were characterized by the ratio of the mass spectrometry signal values of the intracellular metabolites to those of the isotope internal standards labeled with ^13^C. The results for organic acids within the central carbon metabolism were shown in Fig. 4. Significantly, pyruvate showed opposite trends against organic acids in the TCA cycle. The addition of vitamins reduced the intracellular concentration of pyruvate but increased the levels of organic acids in TCA cycle. Pyruvate dehydrogenase was the key enzyme that catalyzed the conversion of pyruvate to acetyl-CoA, and TPP was an important coenzyme for this enzyme (Bettendorff and Wins 2009). The exogenous addition of TPP might enhance the conversion capacity of pyruvate in E3, allowing more flux towards the TCA during the production phase, thereby enhancing the supply of energy and precursor substances required for erythromycin synthesis.

The results of the relative intracellular amino acid concentrations were shown in the Fig. S4. It could be observed that both cysteine and methionine intracellular concentrations in the experimental group were higher than those in the control group during both the cell growth phase and the stationary phase. At the same time, as the most important precursor sources for cysteine and methionine, the content of aspartic acid in the experimental group was significantly lower than that in the control group at 72 h. Tetrahydrofolate, the active form of VB_9_, and VB_12_ acted as a methyl donor and coenzyme, respectively, potentially facilitating the metabolic conversion of aspartic acid into methionine. A higher intracellular level of methionine can promote the synthesis of S-adenosylmethionine (SAM), which would be beneficial to the methyl supply during erythromycin synthesis and is considered to promote the synthesis of erythromycin A (Haydock et al. 1991). However, considering that the size of the intracellular metabolite pool was simultaneously regulated by multiple factors, changes in the concentration of intracellular organic acids might be the result of a comprehensive impact of their upstream and downstream metabolism. The specific metabolic mechanism needs to be further explored and verified through enzyme activity analysis.

### Metabolic flux analysis exposed the influence of vitamins on central carbon metabolism

The comparative analysis of metabolic fluxes aimed to reveal the potential mechanisms by which vitamins addition influenced the metabolic pathways, consequently impacting erythromycin biosynthesis. The comprehensive core metabolic network model of *S. erythraea*, encompassing substrate utilization, central carbon metabolism, and product synthesis, had been constructed (Table S3). Utilizing metabolic parameters about substrate consumption and product formation throughout the fermentation, the carbon balance analysis of E3 with the presence or absence of vitamins was performed at various fermentation phases (Table S4). The middle phase of erythromycin fermentation (84–96 h) with high carbon recovery rate was selected for detailed metabolic flux analysis.

**Fig. 5:**
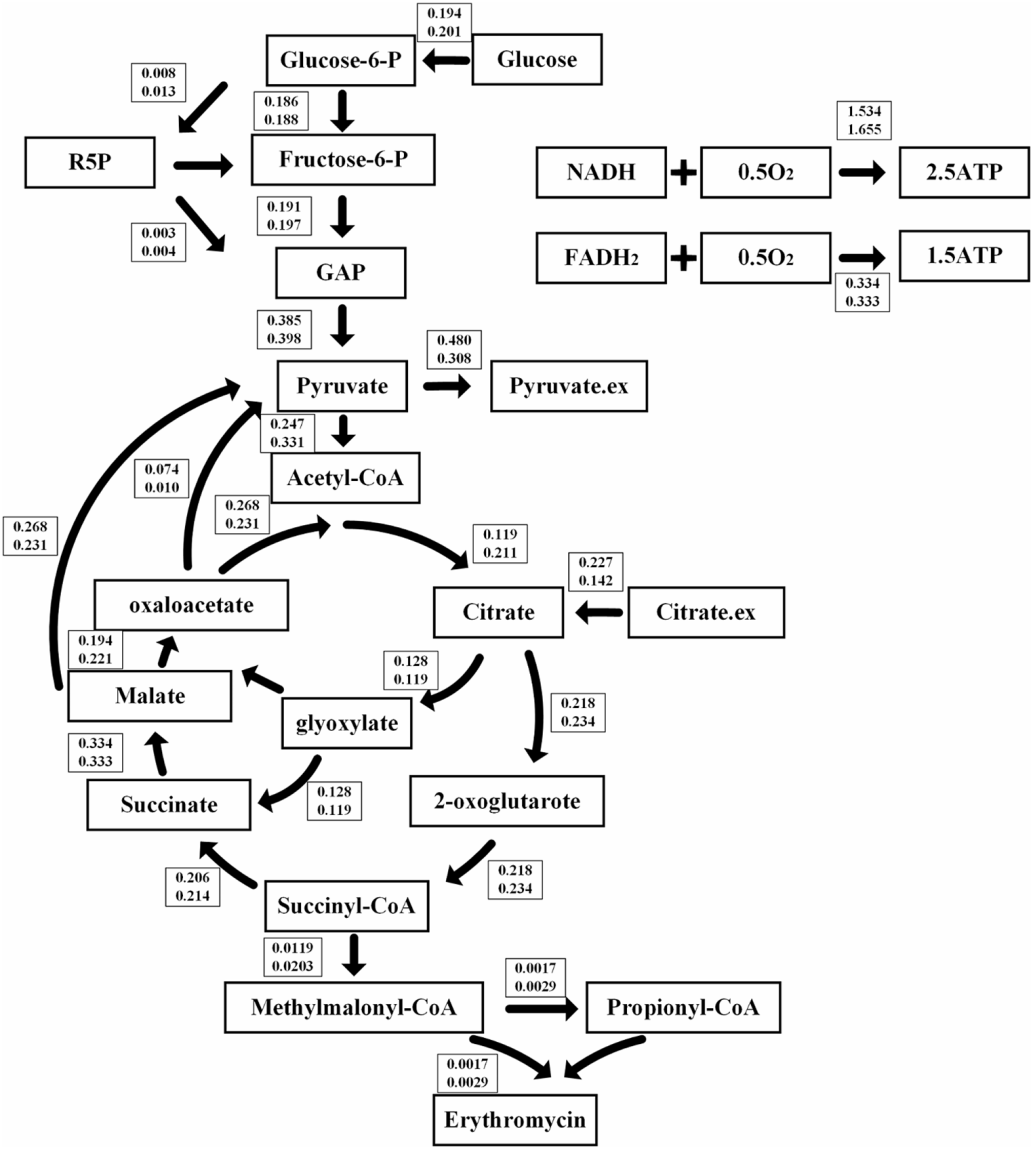
Distribution of carbon metabolism fluxes between *S. erythraea* E3 and vitamins supplemented E3. The above figures indicate the metabolic flux distribution of the vitamins supplemented group, and the numbers below indicate the metabolic flux distribution of the control group. The unit of metabolic flux is mmol/gDCW/h

Under comparable glucose uptake rates, the fluxes through the glycolysis pathway were equivalent between the experimental group and the control group (Fig. 5). The pentose phosphate pathway (PPP) in the experimental group exhibited a modest increase over the control, which is advantageous for cofactor provision during erythromycin synthesis. Post-vitamin supplementation, there was a notable enhancement in the metabolic flux of the TCA cycle compared to the control, with an increased carbon flow into the TCA cycle via pyruvate metabolism. These predictions were corroborated by metabolomics analysis. Notably, there was a significant increase in the metabolic flux from succinyl-CoA to methylmalonyl-CoA. Given that VB_12_ served as an essential coenzyme for methylmalonyl-CoA mutase, the exogenous supplementation of VB_12_ was likely to augment this metabolic pathway (Froese et al. 2019). Cofactor analysis revealed that the experimental group exhibited a higher conversion rate of NADH to ATP, with a sufficient ATP supply being conducive to erythromycin biosynthesis.

## Conclusion

Through comparative transcriptomics, we identified a significant upregulation of genes involved in cofactor and vitamin metabolism. Our experimental approach to individual and combined vitamin supplementation yielded insightful results. Notably, TPP, VB_2_, VB_6_, VB_9_, VB_12_, and hemin were found to positively influence erythromycin synthesis, resulting in a notable increase in the yield of erythromycin. Targeted metabolomics analysis and metabolic flux analysis provided a deeper understanding of the micro-level metabolic changes induced by vitamin supplementation. The results had practical implications for the optimization of industrial fermentation processes and provided a foundation for further research into the metabolic engineering of antibiotic production.

## Electronic supplementary material

Supplementary Material 1

## Data Availability

The authors confirm that the data supporting the findings of this study are available within the article.

## Abbreviations

TPP: Thiamine Pyrophosphate
TCA: Tricarboxylic Acid
CoA: Coenzyme A
KEGG: Kyoto Encyclopedia Of Genes And Genomes
GC-MS: Gas Chromatography-Mass Spectrometry
AMCTB: Analytical Methods Committee Technical Briefs
FMN: Flavin Mononucleotide
FAD: Flavin Adenine Dinucleotide
PBD: Plackett-Burman Design
DCW: Dry Cell Weight
CER: Carbon Emission Rate
IDMS: Isotope Dilution Mass Spectrometry
SAM: S-Adenosylmethionine
PPP: Pentose Phosphate Pathway

## References

[CR22] Averianova LA, Balabanova LA, Son OM, Podvolotskaya AB, Tekutyeva LA (2020) Production of vitamin B2 (Riboflavin) by microorganisms: an overview. Front Bioeng Biotechnol 8:570828. 10.3389/fbioe.2020.570828 33304888 10.3389/fbioe.2020.570828 PMC7693651

[CR21] Bettendorff L, Wins P (2009) Thiamin diphosphate in biological chemistry: new aspects of thiamin metabolism, especially triphosphate derivatives acting other than as cofactors. FEBS J 276(11):2917–2925. 10.1111/j.1742-4658.2009.07019.x 19490098 10.1111/j.1742-4658.2009.07019.x

[CR4] Bu QT, Li YP, Xie H, Li JF, Lv ZY, Su YT, Li YQ (2021) Rational engineering strategies for achieving high-yield, high-quality and high-stability of natural product production in actinomycetes. Metab Eng 67:198–215. 10.1016/j.ymben.2021.06.003 34166765 10.1016/j.ymben.2021.06.003

[CR13] Depeint F, Bruce WR, Shangari N, Mehta R, O’Brien PJ (2006) Mitochondrial function and toxicity: role of the B vitamin family on mitochondrial energy metabolism. Chem Biol Interact 163(1–2):94–112. 10.1016/j.cbi.2006.04.014 16765926 10.1016/j.cbi.2006.04.014

[CR26] Froese DS, Fowler B, Baumgartner MR (2019) Vitamin B(12), folate, and the methionine remethylation cycle-biochemistry, pathways, and regulation. J Inherit Metab Dis 42(4):673–685. 10.1002/jimd.12009 30693532 10.1002/jimd.12009

[CR6] Guo Q, Chu J, Zhuang Y, Gao Y (2016) Controlling the feed rate of propanol to optimize erythromycin fermentation by on-line capacitance and oxygen uptake rate measurement. Bioprocess Biosyst Eng 39(2):255–265. 10.1007/s00449-015-1509-1 26615414 10.1007/s00449-015-1509-1

[CR30] Haydock SF, Dowson JA, Dhillon N, Roberts GA, Cortes J, Leadlay PF (1991) Cloning and sequence analysis of genes involved in erythromycin biosynthesis in *Saccharopolyspora erythraea*: sequence similarities between EryG and a family of S-adenosylmethionine-dependent methyltransferases. Mol Gen Genet 230(1–2):120–128. 10.1007/BF00290659 1840640 10.1007/BF00290659

[CR17] Hong M, Mou H, Liu X, Huang M, Chu J (2017) (13)C-assisted metabolomics analysis reveals the positive correlation between specific erythromycin production rate and intracellular propionyl-CoA pool size in *Saccharopolyspora erythraea*. Bioprocess Biosyst Eng 40(9):1337–1348. 10.1007/s00449-017-1792-0 28567527 10.1007/s00449-017-1792-0

[CR14] Huang HJ, Liu LM, Li Y, Du GC, Chen J (2006) Redirecting carbon flux in *Torulopsis glabrata* from pyruvate to alpha-ketoglutaric acid by changing metabolic co-factors. Biotechnol Lett 28(2):95–98. 10.1007/s10529-005-4953-1 16369692 10.1007/s10529-005-4953-1

[CR8] Jiang M, Pfeifer BA (2013) Metabolic and pathway engineering to influence native and altered erythromycin production through *E. Coli*. Metab Eng 19:42–49. 10.1016/j.ymben.2013.05.005 23747605 10.1016/j.ymben.2013.05.005

[CR27] Layer G (2021) Heme biosynthesis in prokaryotes. Biochim Biophys Acta Mol Cell Res 1868(1):118861. 10.1016/j.bbamcr.2020.118861 32976912 10.1016/j.bbamcr.2020.118861

[CR28] León-Del-Río A (2019) Biotin in metabolism, gene expression, and human disease. J Inherit Metab Dis 42(4):647–654. 10.1002/jimd.12073 30746739 10.1002/jimd.12073

[CR11] Li YY, Chang X, Yu WB, Li H, Ye ZQ, Yu H, Liu BH, Zhang Y, Zhang SL, Ye BC, Li YX (2013) Systems perspectives on erythromycin biosynthesis by comparative genomic and transcriptomic analyses of *S. Erythraea* E3 and NRRL23338 strains. BMC Genomics 14(1):523. 10.1186/1471-2164-14-523 23902230 10.1186/1471-2164-14-523 PMC3733707

[CR19] Li X, Ke X, Qiao L, Sui Y, Chu J (2022) Comparative genomic and transcriptomic analysis guides to further enhance the biosynthesis of erythromycin by an overproducer. Biotechnol Bioeng 119(6):1624–1640. 10.1002/bit.28059 35150130 10.1002/bit.28059

[CR15] Liao Z, Suo Y, Xue C, Fu H, Wang J (2018) Improving the fermentation performance of *Clostridium acetobutylicum* ATCC 824 by strengthening the VB1 biosynthesis pathway. Appl Microbiol Biotechnol 102(18):8107–8119. 10.1007/s00253-018-9208-x 29987383 10.1007/s00253-018-9208-x

[CR29] Lyon P, Strippoli V, Fang B, Cimmino L (2020) B vitamins and one-Carbon Metabolism: implications in Human Health and Disease. Nutrients 12(9). 10.3390/nu12092867 10.3390/nu12092867 PMC7551072 32961717

[CR9] Oliynyk M, Samborskyy M, Lester JB, Mironenko T, Scott N, Dickens S, Haydock SF, Leadlay PF (2007) Complete genome sequence of the erythromycin-producing bacterium *Saccharopolyspora erythraea* NRRL23338. Nat Biotechnol 25(4):447–453. 10.1038/nbt1297 17369815 10.1038/nbt1297

[CR1] Palazzotto E, Tong Y, Lee SY, Weber T (2019) Synthetic biology and metabolic engineering of actinomycetes for natural product discovery. Biotechnol Adv 37(6):107366. 10.1016/j.biotechadv.2019.03.005 30853630 10.1016/j.biotechadv.2019.03.005

[CR5] Park JW, Yoon YJ (2019) Recent advances in the discovery and combinatorial biosynthesis of microbial 14-membered macrolides and macrolactones. J Ind Microbiol Biotechnol 46(3–4):445–458. 10.1007/s10295-018-2095-4 30415291 10.1007/s10295-018-2095-4

[CR10] Peano C, Damiano F, Forcato M, Pietrelli A, Palumbo C, Corti G, Siculella L, Fuligni F, Tagliazucchi GM, Benedetto GED, Bicciato S, Bellis GD, Alifano P (2014) Comparative genomics revealed key molecular targets to rapidly convert a reference rifamycin-producing bacterial strain into an overproducer by genetic engineering. Metab Eng 26:1–16. 10.1016/j.ymben.2014.08.001 25149266 10.1016/j.ymben.2014.08.001

[CR24] Phillips RS (2015) Chemistry and diversity of pyridoxal-5’-phosphate dependent enzymes. Biochim Biophys Acta 1854(9):1167–1174. 10.1016/j.bbapap.2014.12.028 25615531 10.1016/j.bbapap.2014.12.028

[CR2] Redenbach M, Scheel J, Schmidt U (2000) Chromosome topology and genome size of selected actinomycetes species. Antonie Van Leeuwenhoek 78(3):227–235. 10.1023/A:1010289326752 11386344 10.1023/a:1010289326752

[CR23] Rosenberg J, Ischebeck T, Commichau FM (2017) Vitamin B6 metabolism in microbes and approaches for fermentative production. Biotechnol Adv 35(1):31–40. 10.1016/j.biotechadv.2016.11.004 27890703 10.1016/j.biotechadv.2016.11.004

[CR25] Selhub J (2002) Folate, vitamin B12 and vitamin B6 and one carbon metabolism. J Nutr Health Aging 6(1):39–42 11813080

[CR20] Shanbhag AP (2019) FabG: from a core to circumstantial catalyst. Biotechnol Lett 41(6–7):675–688. 10.1007/s10529-019-02678-2 31037463 10.1007/s10529-019-02678-2

[CR12] Wang M, Chen B, Fang Y, Tan T (2017) Cofactor engineering for more efficient production of chemicals and biofuels. Biotechnol Adv 35(8):1032–1039. 10.1016/j.biotechadv.2017.09.008 28939499 10.1016/j.biotechadv.2017.09.008

[CR3] Weber T, Charusanti P, Musiol-Kroll EM, Jiang X, Tong Y, Kim HU, Lee SY (2015) Metabolic engineering of antibiotic factories: new tools for antibiotic production in actinomycetes. Trends Biotechnol 33(1):15–26. 10.1016/j.tibtech.2014.10.009 25497361 10.1016/j.tibtech.2014.10.009

[CR18] Xu F, Ke X, Hong M, Huang M, Chen C, Tian X, Hang H, Chu J (2021) Exploring the metabolic fate of propanol in industrial erythromycin-producing strain via 13 C labeling experiments and enhancement of erythromycin production by rational metabolic engineering of *Saccharopolyspora erythraea*. Biochem Biophys Res Commun 542:73–79. 10.1016/j.bbrc.2021.01.024 33497965 10.1016/j.bbrc.2021.01.024

[CR7] You D, Wang MM, Yin BC, Ye BC (2019) Precursor supply for Erythromycin Biosynthesis: Engineering of Propionate Assimilation Pathway based on Propionylation Modification. ACS Synth Biol 8(2):371–380. 10.1021/acssynbio.8b00396 30657660 10.1021/acssynbio.8b00396

[CR16] Yu G, Jia X, Wen J, Wang G, Chen Y (2011) Enhancement of daptomycin production in *Streptomyces roseosporus* LC-51 by manipulation of cofactors concentration in the fermentation culture. World J Microbiol Biotechnol 27(8):1859–1868. 10.1007/s11274-010-0644-8

