# Supplementary material for: Transcriptomics-guided optimization of vitamins to enhance erythromycin yield in saccharopolyspora erythraea
Source: Bioresour Bioprocess. 2024 Nov 1;11(1):105. doi: 10.1186/s40643-024-00817-w (PMC11530413; doi:10.1186/s40643-024-00817-w)
Supplement: Supplementary file 1 — Supplementary Material 1 [file 40643_2024_817_MOESM1_ESM.docx]

**Transcriptomics-guided optimization of vitamins to enhance erythromycin yield in *saccharopolyspora erythraea***

**Xiang Ke^1^, Xing Jiang^1^, Shuohan Wang^1^, Xiwei Tian^1, 2^* and Ju Chu^1^***

^1^ State Key Laboratory of Bioreactor Engineering, East China University of Science and Technology, Shanghai 200237, China

^2^ Qingdao Innovation Institute of East China University of Science and Technology, 596-1 East Jiushui Road, Qingdao, 266102, China

*Correspondence:

Xiwei Tian: xiweitian@ecust.edu.cn; Tel. (+86) 021-64257019; 130 Meilong Road, Shanghai 200237, China.

Ju Chu: juchu@ecust.edu.cn; Tel. (+86) 021-64253021; 130 Meilong Road, Shanghai 200237, China.

**Supplemental materials**

**Table S1** Concentration levels of vitamins used in the Plackett-Burman experimental design

|  | Factors | High level (mg/L) | Low level (mg/L) |
| --- | --- | --- | --- |
| F1 | TPP | 0.75 | 0.25 |
| F2 | VB2 | 0.5625 | 0.1875 |
| F3 | VB6 | 0.45 | 0.15 |
| F4 | VB7 | 7.5 | 2.5 |
| F5 | VB12 | 0.15 | 0.45 |
| F6 | Hemin | 2 | 6 |

**Table S2** The design of Plackett-Burman experiment

| RUN | F1 | F2 | F3 | F4 | F5 | F6 |
| --- | --- | --- | --- | --- | --- | --- |
| 1 | -1 | -1 | -1 | 1 | -1 | 1 |
| 2 | 1 | 1 | 1 | -1 | -1 | -1 |
| 3 | -1 | -1 | 1 | 1 | 1 | 1 |
| 4 | 1 | -1 | 1 | 1 | 1 | -1 |
| 5 | -1 | 1 | 1 | 1 | -1 | -1 |
| 6 | -1 | -1 | -1 | 1 | -1 | -1 |
| 7 | 1 | -1 | 1 | 1 | -1 | 1 |
| 8 | 1 | 1 | -1 | 1 | -1 | 1 |
| 9 | 1 | 1 | -1 | 1 | 1 | 1 |
| 10 | -1 | 1 | -1 | 1 | 1 | -1 |
| 11 | 1 | -1 | 1 | 1 | 1 | -1 |
| 12 | 0 | 0 | 0 | 0 | 0 | 0 |
| 13 | -1 | 1 | 1 | 1 | 1 | 1 |

**Table S3** The central carbon metabolic network model

| No. | Reaction |
| --- | --- |
| r1 | Glc+ATP → G6P |
| r2 | G6P → F6P |
| r3 | G6P → R5P+2·NADPH+CO_2_ |
| r4 | 3·R5P → 2·F6P+GAP |
| r5 | F6P+ATP→2·GAP |
| r6 | GAP→Pyr+2·ATP+NADH |
| r7 | Pyr →AcCoA + NADH + CO_2_ |
| r8 | AcCoA+OAC → Icit |
| r9 | Cit → α-KG+NADH+CO_2_ |
| r10 | α-KG → SucCoA+NADH +CO_2_ |
| r11 | SucCoA → OAC+NADH+FADH_2_+ATP |
| r12 | Suc → Mal + FADH_2_ |
| r13 | Mal → OAC + NADH |
| r14 | OAC → Pyr + ATP + CO_2_ |
| r15 | Cit + AcCoA→ Mal + Suc |
| r16 | Mal → Pyr + CO2 + NADH |
| r17 | SucCoA → MetCoA |
| r18 | MetCoA → ProCoA + ATP + CO_2_ |
| r19 | ProCoA + 6·MetCoA + 2·Glc + 9·NADPH→ Ery + 2·NADH + 6·CO_2_ |
| r20 | NADH + 0.5 ·O_2_ → 2.5 ·ATP |
| r21 | FADH_2_ + 0.5·O_2_ → 1.5 ·ATP |
| r22 | Cit → Cit.ex |
| r23 | Pyr → Pyr.ex |
| r24 | CO_2_ → CO_2_.ex |
| r25 | O_2_.ex → O_2_ |
| r26 | Ery → Ery.ex |
| r27 | Glc.ex → Glc |

**Table S4** The specific rate and carbon recovery rate of E3 fermentation between 84-96 h

|  | E3 | E3 with vitamins |
| --- | --- | --- |
| q_ery_(mmol/gDCW/h) | 0.00168 | 0.00293 |
| q_CO2_(mmol/gDCW/h) | 1.046 | 1.0732 |
| q_O2_(mmol/gDCW/h) | 0.933 | 0.994 |
| q_glu_(mmol/gDCW/h) | 0.196 | 0.207 |
| *Carbon recovery rate（%） | 93.9% | 95.0% |

*Carbon recovery rate（%）=（q_ery_×37+q_co2_×1）/（q_glucose_×6）


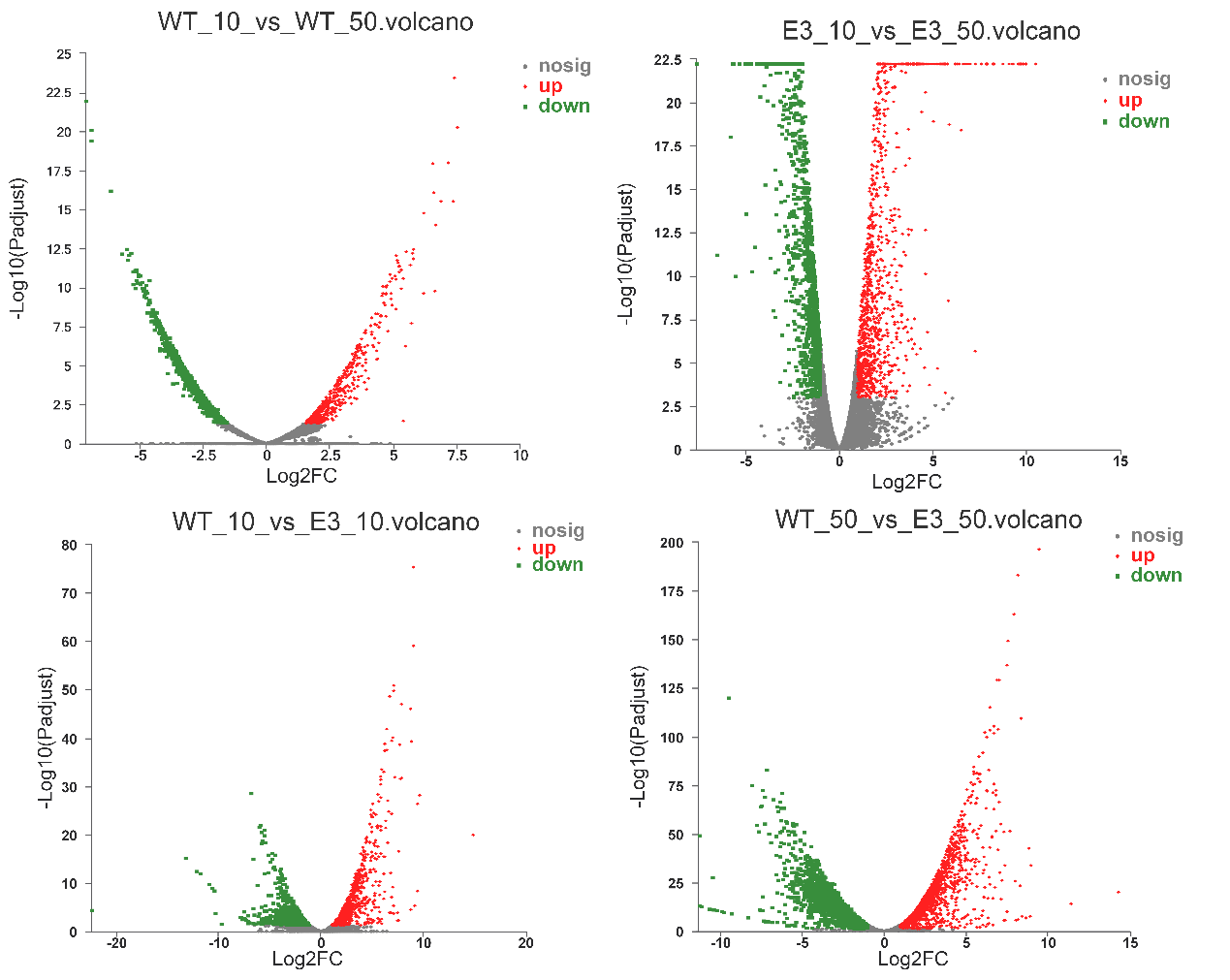


Fig.S1 Volcano plot of gene expression differences between *S. erythraea* NRRL2338 (WT) and E3 at different phase. The horizontal axis represents the fold change (FC) value, which indicates the magnitude of gene expression difference between two sample groups. The vertical axis represents the statistical significance of the difference in gene expression, p-value.


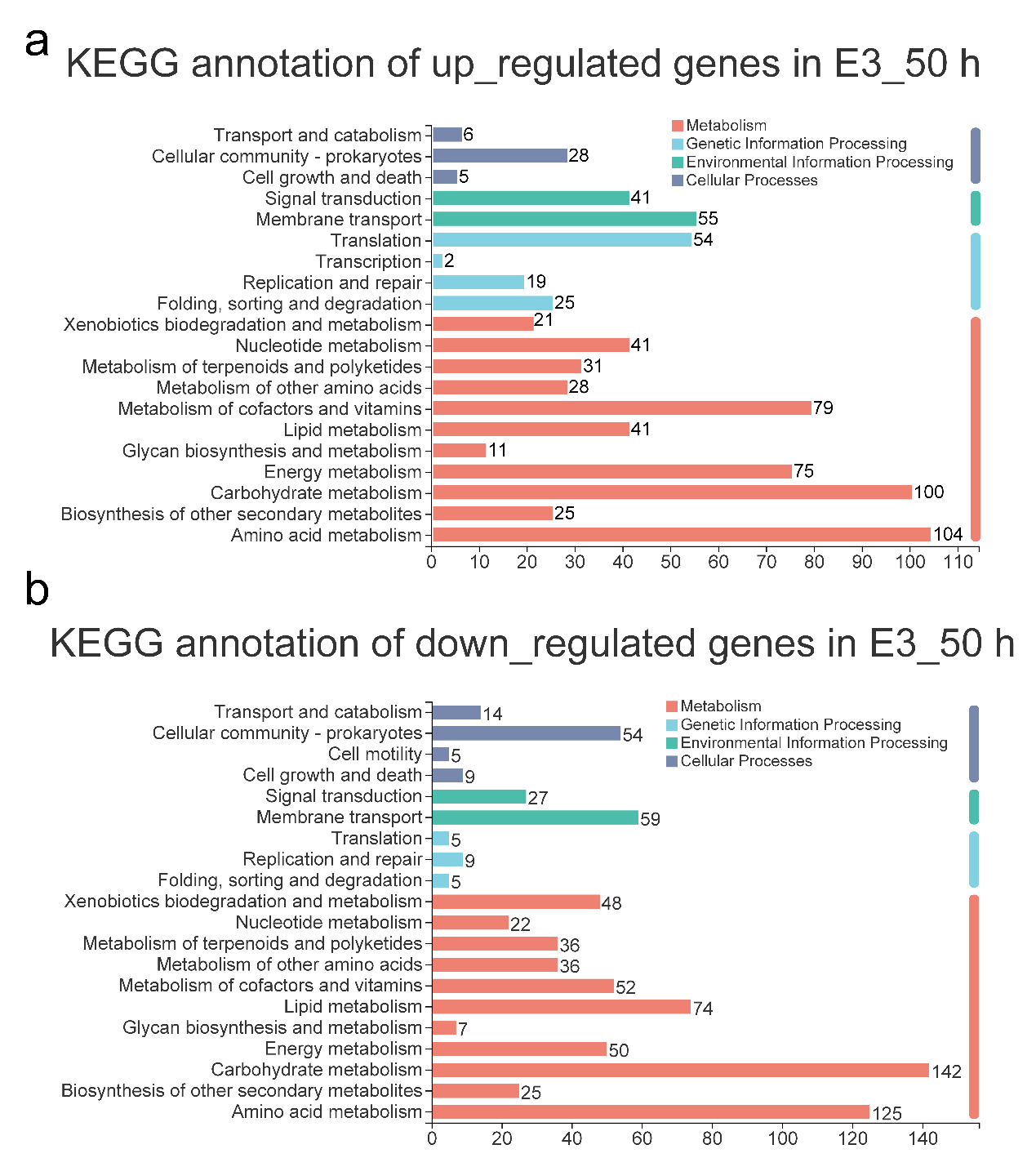


**Fig.S2** KEGG Annotation of specific differential expression genes in E3_50 h


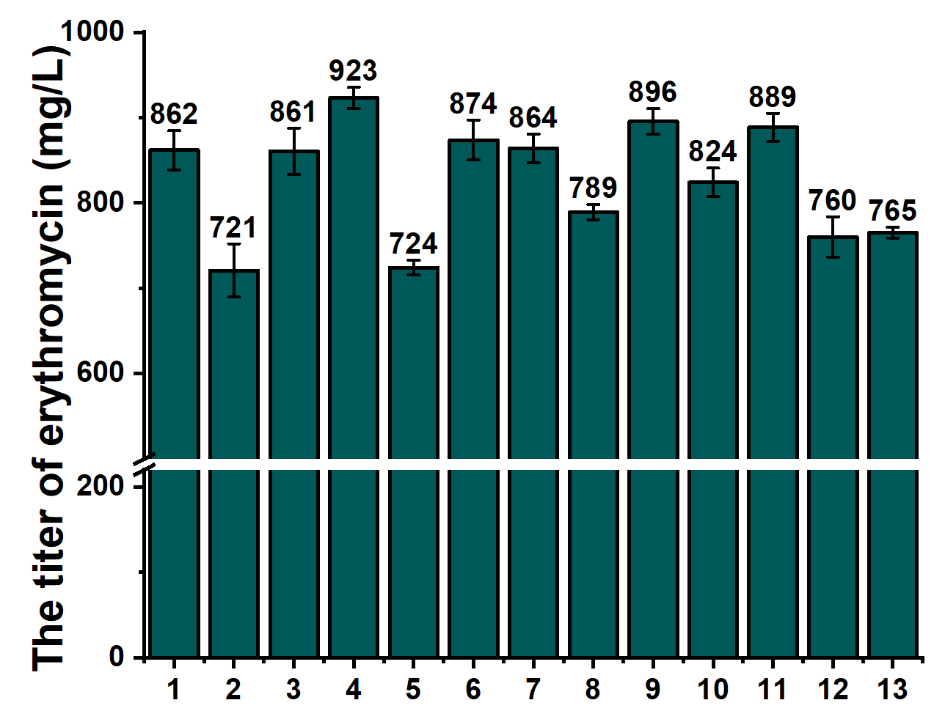


**Fig. S3** The response values of Plackett-Burman experiment


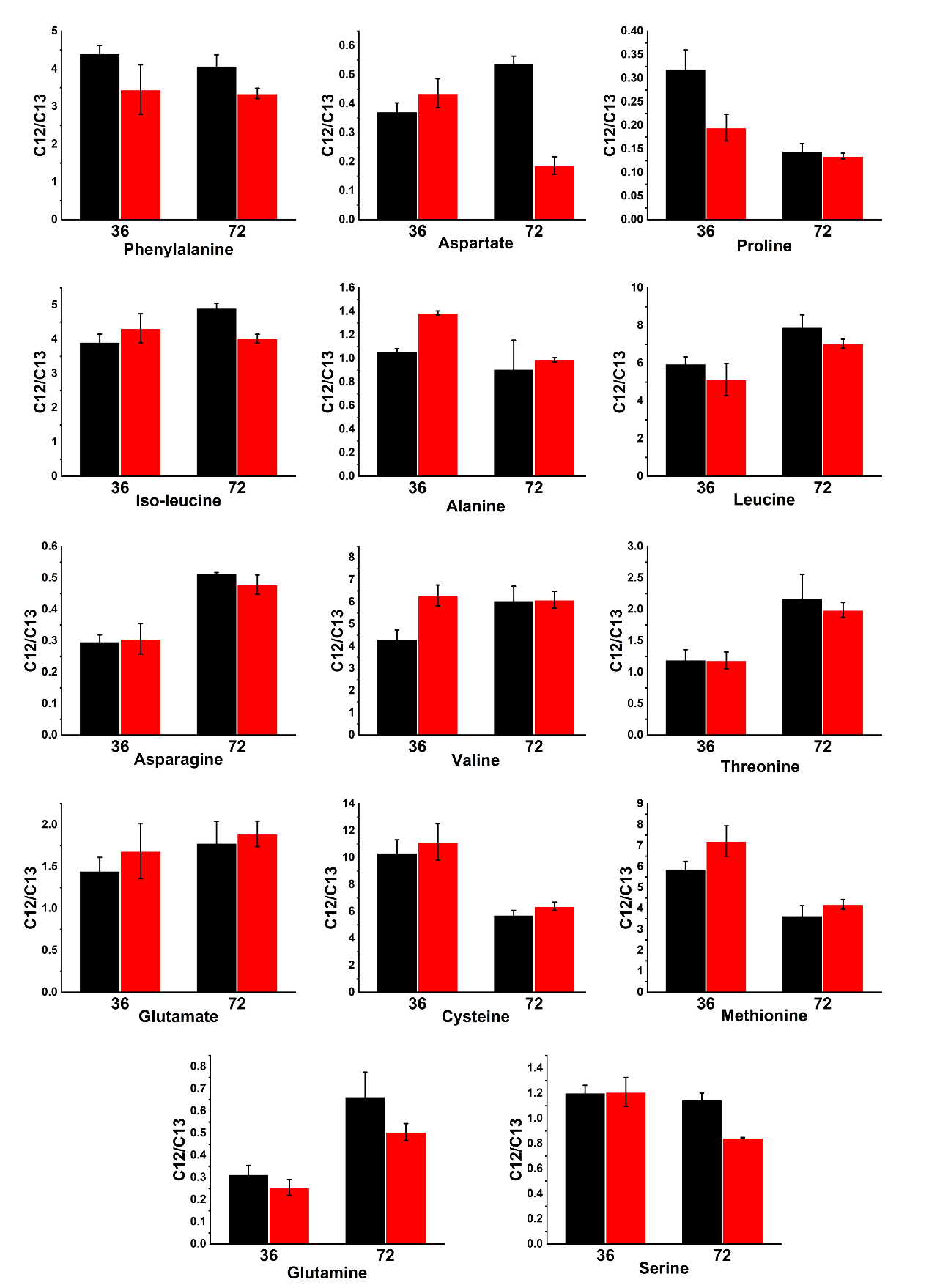


**Fig.S4** The effects of vitamins supplementation on the relative intracellular concentrations of amino acids in E3 at 36 h and 72 h. The relative concentration of intracellular organic acids is represented by the ratio of ^12^C intracellular metabolites to ^13^C internal standards
